# Potential Role of Sugar Transporters in Cancer and Their Relationship with Anticancer Therapy

**DOI:** 10.1155/2010/205357

**Published:** 2010-07-18

**Authors:** Moisés Blanco Calvo, Angélica Figueroa, Enrique Grande Pulido, Rosario García Campelo, Luís Antón Aparicio

**Affiliations:** ^1^Biomedical Research Institute, A Coruña University Hospital, As Xubias 84, 15006 A Coruña, Spain; ^2^Clinical Oncology Department, Ramón y Cajal University Hospital, Ctra. de Colmenar Viejo Km. 9,100, 28034 Madrid, Spain; ^3^Clinical Oncology Department, A Coruña University Hospital, As Xubias 84, 15006 A Coruña, Spain; ^4^Medicine Department, University of A Coruña, Oza s/n, 15006 A Coruña, Spain

## Abstract

Sugars, primarily glucose and fructose, are the main energy source of cells. Because of their hydrophilic nature, cells use a number of transporter proteins to introduce sugars through their plasma membrane. Cancer cells are well known to display an enhanced sugar uptake and consumption. In fact, sugar transporters are deregulated in cancer cells so they incorporate higher amounts of sugar than normal cells. In this paper, we compile the most significant data available about biochemical and biological properties of sugar transporters in normal tissues and we review the available information about sugar carrier expression in different types of cancer. Moreover, we describe the possible pharmacological interactions between drugs currently used in anticancer therapy and the expression or function of facilitative sugar transporters. Finally, we also go into the insights about the future design of drugs targeted against sugar utilization in cancer cells.

## 1. Introduction

In animal cells, sugars are the major source of metabolic energy. However, as the plasma membrane is impermeable to polar molecules, membrane-associated carrier proteins are necessary for the introduction of sugars in cells. There are two described families of transporters: GLUT [solute carrier family 2 (facilitated glucose transporter), gene name SLC2A] and SGLT [solute carrier family 5 (sodium/glucose cotransporter); gene name: SLC5A]. GLUT transporters use existing gradients in sugar concentration, between external and internal sides of plasma membrane, to facilitate its translocation. Conversely, SGLT proteins move sugars inside of cells against gradient concentration, with the consequent energy cost. There are distinct *GLUT* genes encoding distinct GLUT transporters, which share an important sequence homology, although they display different affinity for sugars and present a marked tissue-specific expression pattern. Among all sugars, glucose is the most used by cells. Glucose uptake is the rate-limiting step in its use, underlining the importance of GLUT transporters in metabolism.

Glycolysis is the catabolic pathway by which glucose undergoes the first transformation to obtain energy. In normal cells, after glycolysis, glucose is further metabolized through tricarboxilic acid cycle and oxidative phosphorylation in mitochondria. However, cancer cells display an increased consumption of glucose [1] which is metabolized primarily through the fermentative pathway with the consequent lactic acid production [1, 2]. Probably, the use of fermentative catabolism by cancer cells is due to a mitochondrial malfunction [1, 2]. As result, the oxidative catabolism, which is more efficient in energy production, is impaired in cancer cells. The fermentation pathway carried out by cancer cells implies the consumption of more sugar to fulfill their energy requirements. Indeed, distinct studies have shown the glucose transporter induction during malignant transformation [3]. In cancer cells, it has been also proposed an enhanced activity of glycolytic enzymes, especially the activity of the enzyme hexokinase, which phosphorylates glucose to avoid its exit from the cell and to maintain its transmembrane gradient.

In this paper, we reviewed data available about the normal behavior of sugar transporters, as well as their expression in malignant lesions and their possible pharmacological modulation by new anticancer drugs.

## 2. Expression and Roles of Sugar Transporters in Normal Tissues

### 2.1. Sugar Transporters

Sugars are primarily obtained from the diet after the hydrolysis of disaccharides and polysaccharides, although it is also possible that their synthesis can happen in organs such as liver. The dietary sugars are captured by enterocytes that coat the lumen of small intestine. In addition, both dietary and synthesized sugars must be transferred to blood for their transport along organism. This sugar transport is performed through sugar transporter proteins, located at plasma membrane of cells. The location of sugar transporters is intracellularly regulated by polarity, especially in enterocytes, where each side (apical-luminal or basolateral) of cell displays specific transporters with different features. In addition, sugar transporters show also a tissue-specific expression, and this expression reflects the physiological characteristics of each tissue in relation to sugar transporter features. There are two types of sugar transporters depending on their use of energy for sugar transport: Na^+^-dependent sugar cotransporters (SGLT), which require energy for sugar transport, and facilitative Na^+^-independent sugar transporters (GLUT), which utilize sugar concentration gradient to move it through membranes.

### 2.2. Sodium-Dependent Sugar Transporters

The family of SGLT transporters comprises the sodium-glucose symporters SGLT1 and SGLT2, the glucose sensor SGLT3, the multivitamin transporters SGLT4 and SGLT6, as well as the thyroid iodide transporter SGLT5. Their predicted secondary structures contain fourteen transmembrane helices with their N-terminus and C-terminus regions orientated to the extracellular face. SGLT1 and SGLT2 are carrier proteins that move glucose and also galactose with much lower affinity. They transport these sugars across the plasma membrane against gradient concentration, with the consequent energy expenditure. SGLT1 and SGLT2 proteins are able to exchange sugars with sodium ions through the membrane to carry out this transport. The sodium electrochemical gradient necessary to this transport is generated by the Na^+^-K^+^ pump, which uses ATP to transport sodium against its gradient concentration. SGLT1 expression is found essentially restricted at the apical membranes of enterocytes from small intestine and cells from renal proximal tubules (S3 cells). SGLT2 is expressed predominantly on apical membranes of cells from renal convoluted proximal tubules (S1 and S2 cells). SGLT2 shows low affinity and high capacity to transport glucose while SGLT1 displays high affinity and low capacity of transport. Thus, in kidney, SGLT2 is responsible for the recovery of the bulk of plasma glucose from glomerular filtrate, while SGLT1 is responsible for recuperation of the remaining glucose, avoiding its loss in urine [4, 5].

### 2.3. Facilitative Sugar Transporters

Facilitative sugar transporters, GLUTs, contrary to SGLTs, move sugars (including glucose, fructose, and other hexoses) across cell membrane, without energy consumption and in favor of gradient concentration. To date, it has been identified fourteen GLUTs [4–6], grouped in three classes, depending on their structural and sequence similarity. However, there is a high degree of homology between these GLUT transporters, which share common features: they have twelve transmembrane domains with intracellular carboxyl- and amino-ends and display conserved glycine and tryptophan residues, which may be essential for their function [5]. The most remarkable difference between class I and II, and class III members is the position of a predicted long extracellular loop. This loop, in members of class I and II, is located between transmembrane domains 1 and 2, and contains a glycosylation site which appears to modulate their capacity of transport. The class III members contain this extracellular loop with their glycosylation sites between transmembrane domains 9 and 10 [5–7]. The model that explains the movement of sugar by GLUTs is based on the adoption of two exclusive alternative conformations of transporters: in one conformation GLUTs expose a binding site for sugar to extracellular side of plasma membrane, while in the second conformation GLUTs expose this binding site to the intracellular side of plasma membrane. The binding of glucose (or other compatible sugars) to one of these two sites triggers a conformational change of GLUT transporters to one or other conformation. In this process, sugars are moved across plasma membrane in any of the two directions [6]. However, despite similarities, GLUTs display different capabilities to transport distinct sugars and they also present different regulation and different distribution among tissues. In particular, GLUT tissue-specific expression (Table 1) may play important roles in the regulation of glucose uptake and its metabolism, under distinct nutritional and hormonal conditions [4, 5].

### 2.4. Class I Facilitative Sugar Transporters

Class I of facilitative sugar transporters is formed by GLUT1-4 and GLUT14 [4]. GLUT1 was the first facilitative sugar transporter discovered, and is able to transport primarily glucose, although it can also move galactose, mannose, and glucosamine with distinct efficiencies. GLUT1 is responsible for basal glucose uptake required to maintain respiration in cells, and its expression is usually correlated with the rate of glucose metabolism and respiration. Therefore, although GLUT1 is expressed along virtually all tissues in normal conditions, the highest levels are found in erythrocytes (due to this it is also called erythrocyte-type glucose transporter) and in endothelial cells from blood-tissue barriers, particularly in blood-brain barrier [6]. Nevertheless, recent findings have shown that high GLUT1 expression is not a ubiquitous fact among erythrocytes from all species [8]. Thus, high GLUT1 expression in erythrocytes is restricted to species unable to synthesize vitamin C from glucose, such as in species of primates (including humans), guinea pigs, or fruit bats. Probably, the high expression of GLUT1 in erythrocytes from these species is related to GLUT1-mediated transport of vitamin C during erythroid differentiation [8]. Finally, GLUT1 expression is repressed by p53, an important tumor suppressor in cancer [9]. The alteration in p53 expression may explain GLUT1 overexpression observed in many cancer types, as well as their enhanced glucose metabolism and their higher energy consumption. 

GLUT2 is primarily a glucose transporter (although it displays the highest affinity for glucosamine) that shows low affinity for fructose, galactose, and mannose. GLUT2 is expressed in basolateral membrane of intestinal absorptive epithelium where it is responsible for the transport of fructose and glucose (introduced in enterocytes through SGLT1 which generates a favorable concentration gradient) to blood. In addition, GLUT2 is also expressed in basolateral surface of kidney absorptive cells, where it plays a similar function that in intestine, absorbing glucose from filtrate to blood. In liver, GLUT2 is located in the sinusoidal membrane where it is involved both in blood glucose uptake and in its release. Finally, GLUT2 is also expressed in pancreatic *β*-cells, responsible for insulin synthesis, where it participates in glucose sensing mechanism and, therefore, in the regulation of glucose-stimulated insulin secretion [6].

On the other hand, GLUT3 transports glucose with very high affinity, although it is also able to transport galactose, mannose, maltose, or xylose, but it is unable to transport fructose [6, 10]. GLUT3 is primarily located in tissues with high glucose demand and energy consumption, possibly due to its high affinity for glucose and its greater transport capacity than other GLUT transporters [5], such as GLUT1. Thus, while GLUT3 mRNA is ubiquitously expressed in all human tissues, its protein is primarily located in neurons [6]. GLUT3 transport capacity and its affinity for glucose are particularly important in this cell type, due to the low concentration of glucose in the interneuronal space compared to the concentration in serum. Therefore, while in blood-brain barrier epithelium, GLUT1 (with a lower affinity for glucose than GLUT3) is expressed to transport glucose from blood to interneuronal space, in neurons, GLUT3 introduces this glucose. Indeed, although GLUT1 is expressed in the remaining brain cells, the higher transport capacity and affinity of GLUT3 for glucose enables preferential access to glucose in neurons. On the other hand, GLUT3 has been detected in platelets and in the entire set of white blood cells (lymphocytes, neutrophils, monocytes, and macrophages). Interestingly, in these cells, GLUT3 is located in an intracellular pool from which may be recruited to the plasma membrane under energy needed conditions, similar to GLUT4 translocation in response to insulin, in muscle and adipose tissue. Thus, GLUT3 translocation is promoted by different physiological/signaling events, triggered upon activation of these cells. In B-lymphocytes and monocytes, insulin causes GLUT3 translocation. In platelets, GLUT3 is translocated from intracellular formations, named *α*-granules. This translocation is triggered by thrombin, which induces a number of energy-dependent cell changes producing cell aggregation and clot formation. GLUT3 translocation in neutrophils is triggered by exposure to bacteria (or other specific activators). The activation of neutrophils, monocytes, and lymphocytes involves also a set of changes related to their function and associated to energy expenditure: phagocytosis and elimination of bacteria, immunoglobulin production, and antigen presentation [10]. Finally, GLUT3 has been demonstrated to be essential for survival and for pre- and postimplantation embryo development [10, 11]. 

Another glucose transporter is GLUT4. GLUT4 can also move glucosamine and dehydroascorbic acid. It displays two sequences of internalization responsible for the protein association with an intracellular compartment under basal insulin levels in plasma. When insulin binds to its receptor, GLUT4 is rapidly translocated to plasma membrane, increasing glucose uptake in the cell [6]. This translocation is probably mediated by a putative phosphatidic acid-binding motif, located in the cytoplasmic loop between helices 2 and 3 [12]. In addition, GLUT4 translocation can also be triggered by exercise probably through the activation of insulin-independent AMPK (AMP-activated protein kinase) pathway [6]. In this way, GLUT4 expression is virtually restricted to tissues with a marked insulin- and exercise-dependent glucose transport; that is, heart, adipose tissue, and skeletal muscle [13–15]. Because muscle and fat tissues comprise a large fraction of the body mass, GLUT4 plays a central role in glucose metabolism. Indeed, in rat adipocytes, GLUT4 represents 90% of glucose transporters [15]. In addition, according to its metabolic importance, GLUT4 expression is regulated in a development- and tissue-specific manner [16]. Thus, GLUT4 expression is regulated during muscle cell differentiation. In myoblasts, during alignment step, GLUT4 expression is low and increases during cell fusion [17]. In addition, the timing and magnitude of GLUT4 expression are different in every tissue, controlled by different factors, such as diet and exercise [18]. For example, GLUT4 levels undergo a strong increase in plasma membrane of skeletal myocytes exposed to insulin [19–21]. Finally, GLUT4, similar to GLUT1, displays an interesting connection with cancer, as both transporters are transcriptionally repressed by p53 [9], a tumor suppressor protein important in cell cycle control and apoptosis, processes that are altered usually in cancer.

The last discovered member of class I sugar transporters is GLUT14, which displays a remarkable similarity in gene and protein sequence to GLUT3. Thus, its origin was attributed to the *GLUT3* duplication, occurred recently in evolution (as it has not being found a GLUT14 ortholog in mice). However, in contrast with the virtually ubiquitous distribution of GLUT3, GLUT14 is expressed specifically in testis [22].

### 2.5. Class II Facilitative Sugar Transporters

The class II facultative sugar transporters include GLUT5, GLUT7, GLUT9, and GLUT11. GLUT5 in humans is only capable to transport fructose, with no ability to transport glucose or galactose [6]. GLUT5 expression is primarily located in small intestine (upper jejunum), and in much lesser extent in kidney, skeletal muscle, adipocytes, testes and sperm, and brain. The role of GLUT5 is particularly important in small intestine, where it is located in the apical membrane of enterocytes, and it is responsible for the fructose transport from food. In kidney, GLUT5 is expressed in the apical side of S3 proximal tubule cells, where it is responsible for recapturing fructose from glomerular filtrate [23].

GLUT7 can move both glucose and fructose with the highest affinity among GLUT transporters, whereas it is unable to transport galactose, xylose, or 2-deoxyglucose. GLUT7, like other fructose transporters (GLUT2, GLUT5, and GLUT9), displays an isoleucine close to the end of seventh transmembrane domain, which it is thought to be important for substrate selectivity. Thus, other non-fructose transporters (GLUT1, GLUT3, and GLUT4) display a valine in that location. In GLUT7, the substitution of its isoleucine in this region gives rise to the loss of fructose transport capacity. *GLUT7* mRNA is primarily expressed in small intestine and colon, and in lesser extent in testis and prostate. Importantly, the presence of GLUT7 both in upper (jejunum) and lower (ileum) small intestine as well as in colon allows hypothesizing a role for this transporter in intestinal absorption of sugars. While SGLT1 and GLUT5 transport the bulk of glucose and fructose, respectively, in jejunum and ileum, their concentration is expected to be very low at the end of ileum and in colon. The expression of GLUT7 in these areas, with its extraordinary affinity for both glucose and fructose, could help to capture the remaining glucose and fructose which was not previously taken. As with other sugar transporters, the expression of GLUT7 in intestine is regulated in a substrate-dependent manner, and it is increased when carbohydrate uptake is also increased [24].

Regarding to GLUT9, there is no many data about its transport activity, although it has been suggested that may be a fructose transporter [6, 25]. However, most importantly, GLUT9 is able to transport urate [25]. Thus, polymorphisms and mutations in GLUT9 were correlated with elevated serum uric acid levels and distinct diseases associated with an imbalance in urate homeostasis [26–29]. These diseases affect primarily to organs and locations particularly sensitive to urate imbalance, which match curiously with those locations where GLUT9 is primarily expressed, that is, articular chondrocytes and kidney [25, 30, 31]. Other peculiar feature of GLUT9 protein is the existence of two variants, which are differentially expressed in polarized cells: while an isoform is expressed in apical side, the second isoform is expressed in basolateral side [25]. Finally, other important role for GLUT9 seems to be in relation to glucose sensing in pancreatic *β*-cells, in collaboration with GLUT2 [32]. 

The last member to mention of the class II hexose facilitative transporters is GLUT11, which seems to transport glucose and fructose, but not galactose. GLUT11 displays three splice variants, which, due to the presence of three different first exons, differ in their N-terminal sequence of aminoacids. Interestingly, these three GLUT11 isoforms are expressed in a tissue-specific manner. Indeed, GLUT11a is expressed in heart, skeletal muscle, and kidney; GLUT11b is present in kidney, adipose tissue, and placenta; and GLUT11c is located in heart, skeletal muscle, adipose tissue, and pancreas [33].

### 2.6. Class III Facilitative Sugar Transporters

In general, little is known about the members of class III facilitative sugar transporters, but it includes GLUT6, GLUT8, GLUT10, GLUT12, and HMIT. GLUT6 is a low-affinity glucose transporter, which is predominantly expressed in brain, spleen, and peripheral leukocytes [4]. GLUT8 is able to transport glucose with high affinity, and its transport is inhibited by fructose and galactose. Like GLUT6, GLUT8 contains a sorting motif in its amino-terminal end, which functions as signal to translocate the protein to membranous systems, such as endosomes, lysosomes, or endoplasmic reticulum [34]. GLUT8 is expressed primarily in testis and brain [35]. GLUT10 is able to transport glucose, galactose, and deoxyglucose. Its gene was mapped in chromosome 20, on the type 2 diabetes-linked region [36], although different association studies on distinct populations were unable to correlate type-2 diabetes with any polymorphism in *GLUT10* gene [37–41]. However, mutations in GLUT10 gene were associated with arterial tortuosity syndrome, a disease characterized by tortuosity, elongation, stenosis, and aneurysm formation in arteries due to disruption of elastic fibers in arterial wall. In addition, GLUT10 deficiency is associated with the upregulation of TGF-*β* (tumor growth factor-beta) pathway in arterial wall in Loeys-Dietz syndrome, a disease also characterized by arterial tortuosity and aneurysms [42]. *GLUT10* mRNA was detected in heart, lung, brain, liver, skeletal muscle, pancreas, placenta, and kidney [36]. *GLUT12* gene was identified in MCF-7 breast cancer cells by homology with the insulin-stimulated *GLUT4* gene, although its protein sequence displays more similarity with GLUT10. However, its similarity with *GLUT4* is particularly significant, because, like GLUT4, GLUT12 is intracellularly located in absence of insulin [43]. GLUT12 is expressed in adipose tissue, small intestine, skeletal muscle [43], and placenta [44]. Curiously, in type I oxidative fibers of skeletal muscle, GLUT4 and GLUT12 are predominantly expressed regarding to the remaining GLUT transporters [45]. Indeed, similar to GLUT4, insulin is able to induce the translocation of GLUT12 from an intracellular location to plasma membrane in skeletal muscle [46]. Finally, HMIT [H(+)-myoinositol transporter; solute carrier family-2 (facilitated glucose transporter), member 13; gene name: SLC2A13] is a specific transporter for myoinositol, and its activity is stimulated by the decrease of the extracellular pH [47]. HMIT is predominantly expressed in brain, where it is able to transport IP3 (inositol trisphosphate) and may contribute to different signaling processes related with neuronal function [48].

## 3. Expression of Sugar Transporters in Cancer

Many tumors display high rates of glucose uptake. It has been proposed different hypothesis to explain this exacerbated glucose consumption, including the increase of hexokinase expression [49, 50], the decrease of glucose-6-phosphatase-mediated glucose dephosphorylation [51], and/or the overexpression of sugar transporters [52]. Agreed with this last explanation, GLUT1 overexpression has been observed in many human cancers. In addition, GLUT1 expression levels were inversely correlated with prognosis, as the deregulation of GLUT1 expression may reflect the presence of alterations in different signaling pathways. In fact, elevated levels of glucose uptake, one of the hallmarks of malignant cells, are induced by activated ras or src oncogenes which are key elements in the transduction of multiple signaling pathways [3]. In this regard, it has been recently published that, in colorectal cancer cell lines, mutations in *KRAS* (v-Ki-ras2 Kirsten rat sarcoma viral oncogene homolog) or *BRAF* (v-raf murine sarcoma viral oncogene homolog B1) genes, are able to trigger an overexpression of GLUT1 and an increase of the glucose uptake. Furthermore, the exposition of wild type colorectal cancer cell lines to low levels of glucose contributes to the development of mutations in *KRAS*, which give rise, instead, to the upregulation of GLUT1 and to an increase in glucose uptake [53]. Other important alterations in cancer involved in GLUT1 overexpression affect to *MYC* oncogene expression and to the local hypoxia pathway [54, 55]. Moreover, tumor cells may also express glucose transporters that are not substantially expressed under normal conditions. Below, we will summarize the available information about the expression of sugar transporters in different types of cancer (Table 2). 

Similar to GLUT1, SGLT1 induction is also used by cancer cells to enhance their glucose uptake and their glycolysis, so that cancer cells obtain sufficient energy for maintaining their expansive growth [56]. However, there are few studies about the expression of SGLT transporters in tumors. In a pioneer study, it was demonstrated that the expression or activity of an undefined SGLT cotransporter in HT29 colon cancer cell line is modulated by addition/deprivation of glucose in culture [57]. In a most recent study, the expression of *SGLT1* and *SGLT2* genes was analyzed by RT-PCR in autopsies from normal lung and lung primary tumors together with their metastatic lesions. The *SGLT1* and *SGLT2* expression was found unchanged between lung tumor samples and paired normal lung tissue. By analyzing the metastatic lesions (from liver and lymph nodes) of lung tumors, it was found that the expression of *SGLT2* was significantly higher in metastasis areas than in primary tumors, whereas *SGLT1* expression did not display changes [58]. This study is an interesting approach to the study of *SGLT1* and *SGLT2* expression in lung cancer although the nature of the samples (autopsies) may minimize the significance of the obtained results. Moreover, by using an immunohistochemical approach, the expression of SGLT1 (together with BCL2 and p53) was analyzed in pancreatic cancer to relate the data obtained with different survival parameters. In this study, SGLT1 overexpression was significantly correlated with disease-free survival in pancreatic adenocarcinomas [59]. In addition, high SGLT1 expression in pancreatic primary tumors was correlated with high Bcl-2 expression. This prospective study suggests SGLT1 and Bcl-2 as potential prognostic biomarkers for pancreatic cancer, although it should be validated by a more sensitive technique as qRT-PCR. 

In other study, it has been revealed a surprising link between glucose uptake performed by SGLT1, survival of cancer cells, and EGFR (epidermal growth factor receptor), whose malfunction is involved in many carcinogenetic processes. In this study, authors uncover a new EGFR role in human cancer cells, whereby it is able to maintain glucose uptake by cells through the SGLT1 stabilization, promoted by the EGFR-SGLT1 interaction. Therefore, the EGFR-SGLT1 association-dependent maintenance of intracellular glucose level avoids autophagic cell death, promoting survival of cancer cells [60]. 

Deregulated GLUT expression has been described in many tumor types [61]. In RCC (renal cell carcinoma), the expression of different GLUTs is altered in a histological subtype-specific manner. Thus, in conventional clear cell RCC, GLUT1 expression is increased, while the expression of GLUT4, GLTUT9, and GLUT12 decreases versus the healthy kidney. In papillary RCC, GLUT12 is expressed at lower levels than normal kidney. In chromophobe RCC, GLUT4 expression is increased, while the expression of GLUT2 and GLUT5 decreases. Finally, no changes were observed in oncocytoma RCC subtype in terms of expression of GLUT transporters compared to normal kidney [62]. On the other hand, it was analyzed the GLUT1 expression related to histological subtype and different clinical parameters in RCC. GLUT1 shows higher expression in clear cell RCC versus normal kidney [63], cromophobe RCC [64], and papillary RCC [63, 64]. Despite the GLUT1 expression in clear cell RCC, this data could not be associated with clinicopathological parameters [63, 64], but its expression in this RCC subtype was correlated with the HIF1A (hypoxia-inducible factor 1-alpha) expression [64].

Regarding to prostate tumors, there are few studies of GLUT expression in relation to healthy tissue or to clinical-pathological parameters. It has been studied the role and expression of GLUT1 and GLUT12 in prostate cancer cell lines and tumor and hyperplastic prostate tissue sections through different technical approaches. The mRNA and protein of GLUT1 and GLUT12 were detected in all four prostate carcinoma cell lines assayed. Regarding to the analysis in tissue sections, the expression of GLUT1, but not GLUT12 expression, was detected in benign prostatic hyperplasia. Conversely, in prostatic tumor tissue GLUT12 expression was detected but not GLUT1 expression. It is unknown the relevance and the explanation of this change in the pattern of GLUT1 and GLUT12 expression during prostate tumor progression [65].

In lung cancer, there are also few studies on the alteration in the expression of GLUT transporters during tumor development and their potential role as putative clinical-pathological biomarkers. In the most recent study, it was studied and compared the expression of GLUT transporters between primary tumors with different histology, liver metastasis, and normal lung and liver tissues, obtained from 105 autopsy samples. The expression of GLUT1 was significantly higher in primary lung tumors than in normal lung. In liver metastasis, the GLUT3 and GLUT5 expression was significantly higher than in normal lung tissue and primary lung tumors, while the GLUT1 expression did not show differences while comparing normal lung to primary lung tumors. In addition, GLUT5 expression was significantly higher in metastatic liver lesions than in normal liver, and the expression for GLUT3 showed the same tendency, without reaching signification. GLUT1 expression did not displayed differences between normal and metastatic liver tissue. In conclusion, the expression profile of GLUTs analyzed is different in primary lung tumors and liver metastasis, suggesting an increase in the use of GLUT transporters (through the overexpression of GLUT3 and GLUT5) and, probably an increase in glucose/fructose uptake in metastatic areas [66]. Despite the robust technical approach (RT-PCR) used, the conclusions of this study must be taken with caution as the results obtained with autopsies may differ from the results obtained using biopsies, a type of samples generally utilized for cancer diagnosis. Therefore, from this study, it is difficult to associate the expression of GLUTs analyzed with any clinical-pathological parameter. However, in a previous study, it was analyzed the biological significance of GLUT1 and GLUT3 overexpression on 289 archival biopsies from stage I nonsmall cell lung cancer (NSCLC) patients. During this retrospective study, GLUT1 and GLUT3 overexpression was detected primarily in poorly differentiated and undifferentiated tumors. In addition, the overexpression of GLUT1 and/or GLUT3 was associated with poor survival in all NSCLC patients, but especially in patients with well and moderately differentiated tumors [67]. Therefore, GLUT1 and GLUT3 overexpression may be used as prognostic indicators in stage I nonsmall cell lung cancer patients.

Regarding to GLUT expression in breast cancer, in a preliminary study by immunostaining, GLUT12 expression was detected in invasive and noninvasive breast carcinomas, while it was absent (or with weak staining) in adjacent normal breast tissue [68]. In other studies, GLUT5 expression was also detected both in breast cancer cell lines and breast cancer tissues, while in normal breast tissues GLUT5 expression was absent [69, 70]. In addition, GLUT1 expression was also found in breast tumors and it was possible its association with the invasive ability despite the absence of a clear correlation with prognosis [71, 72]. 

In gastric cancer, GLUT1 expression was assayed in an immunohistochemical study performed on 617 gastric carcinomas and 50 tubular adenomas of stomach. GLUT1 expression was primarily restricted to papillary, tubular, and differentiated adenocarcinoma (low positivity in signet ring cell carcinoma and mucinous adenocarcinoma). GLUT1 expression appears in advanced stages of gastric tumor development and increases with disease progression. Moreover, GLUT1 expression was associated with depth of invasion, lymphatic and venous invasion, lymph node and hepatic metastasis, and carcinoma stage. In addition, the survival of patients with tumors that expressed GLUT1 was significantly shorter than those patients with GLUT1-negative tumors [73]. In a recent study, GLUT1 expression was also detected in pancreas carcinoma, although it was not found significant correlation with any prognostic factor [74]. However, GLUT1 forced overexpression in pancreatic cancer cell lines enhances their invasive capacity through the induction of MMP2 (matrix metalloprotease 2) expression and activity [75]. In addition, it has been demonstrated that GLUT1 expression is responsible for the continuous insulin release in insulinoma patients under hypoglicemia [76, 77]. GLUT1 expression was also retrospectively studied in a cohort of 112 colon carcinoma biopsies. As result of this study, GLUT1 expression was associated with tumor progression and poorer prognosis in colorectal cancer [78]. By immunohistochemistry, the expression of GLUT1 was analyzed in the site of deepest invasion of 152 colorectal cancer samples. The aim of this study was assessing the value of GLUT1 as surrogate biomarker for prognosis and metastatic potential in colorectal cancer. The expression of GLUT1 and Ki-67, as cell proliferation marker, were analyzed in three different zones of tumor samples: the deepest invasive site, the central portion, and the superficial part. In central and superficial part of tumors, no significant differences were detected between GLUT1 expression, Ki-67 expression, and clinicopathological parameters. However, in the deepest invasive site, GLUT1 expression was associated with Ki-67 labeling index. In addition, in patients who underwent curative surgery, the GLUT1 expression at the deepest invasive site was significantly associated with poorer prognosis. Therefore, GLUT1 expression at deepest site of tumor invasion may be used as predictor of poor prognosis in advanced colorectal cancer [79]. Probably, the expression of GLUT1 in deepest zones of colorectal tumors may be related to the degree of hypoxia reached in these zones, since GLUT1 is a HIF1 target [80], which is activated in hypoxic environments. Indeed, in other retrospective study on 49 biopsies from rectal carcinoma patients, the expression of GLUT1 was analyzed in relation to different clinical outcome parameters to asses its potential use as biomarker to detect tumor hypoxia. This semiquantitative study was performed using immunohistochemistry. As result, the GLUT1 overexpression was significantly associated with poorer overall survival (probably due to a poorer metastasis-free survival), although it was not detected significant changes in overall survival between patients suffering GLUT1 positive tumors and patients with GLUT1 negative tumors. In addition, although there was a clear correlation between GLUT1 expression and tumor depth, it was only found a significant association between survival and GLUT1 expression in the deep tumor part. This may be indicative of the relationship existing among poor prognosis, hypoxia, and GLUT1 expression, since a most intense degree of hypoxia is reached in deepest parts of tumors. However, the regulation of GLUT1 by other stimuli hinders the potential clinical use of GLUT1 as a surrogate biomarker for tumor hypoxia [81]. 

Regarding to brain tumors, the expression of GLUT1 and GLUT3 was analyzed in a series of 20 different brain tumors [82]. Although the authors failed to detect GLUT1 immunoreactivity in all brain tumors analyzed (astrocytomas, meningiomas, and gliomas), they could detect GLUT3 immunoreactivity in high grade gliomas. In addition, they demonstrated the induction of *GLUT1* and *GLUT3* mRNAs. While in astrocytomas, *GLUT1* mRNA increased with grade, in meningiomas, *GLUT1* mRNA showed no changes. In gliomas, *GLUT3* mRNA also showed a significant increase correlated with grade, in line with the increase observed also in protein immunoreactivity [82]. Moreover, GLUT3 expression may also be related to malignant transformation in astrocytomas as well as to aberrant neovascularisation in glioblastomas [83]. In fact, GLUT3 was also found upregulated in glioblastoma multiforme [84]. Finally, GLUT5 was also found to be expressed in microglia from human gliomas [85]. 

In head and neck carcinoma, GLUT1 and GLUT3 expression was detected [86, 87], while the GLUT2 and GLUT4 expression was not [87]. The expression of GLUT1 and GLUT3 was analyzed on 38 head and neck carcinomas to determine the biological significance of GLUT overexpression in this type of tumors. *GLUT1* and *GLUT3* gene expression was significantly higher in head and neck tumors than in nontumor adjacent areas and normal tissue. The expression level of GLUT1 gene and protein was correlated with poor survival, clinical stage, and lymph node metastasis, while *GLUT3* gene expression was correlated only with lymph node metastasis. However, GLUT3 protein was not detected in any of the analyzed cases from head and neck carcinoma [86]. According to this evidence, in a previous immunohistochemical study, the expression of GLUT3 protein was not detected in normal mucosa, preneoplastic and neoplastic lesions from head and neck squamous cell carcinoma, while high level of GLUT1 expression was correlated with higher grade of dysplasia. The increased expression of GLUT1 in dysplastic lesions and its sustained expression in tumor samples indicate that alterations in GLUT1 expression occur at early stages during development of head and neck squamous carcinomas [88]. Thus, GLUT1 may be an interesting biomarker to detect preneoplastic lesions and to perform a clinical intervention before the development of head and neck carcinoma. Moreover, it was also performed an analysis on 118 oral squamous cell carcinoma patients to determine the relationship between GLUT1 expression and glucose uptake with overall survival. The analysis showed that it was significant association between GLUT1 overexpression, increased glucose uptake, and poor survival in oral squamous cell carcinoma patients [89]. These data support that GLUT1 could be a good biomarker for prognosis in these patients.

The expression of GLUT1 was also extensively studied in sarcomas. In these tumors, the glucose uptake, measured through FDG (^18^F-Deoxyglucose) signal, correlates with the presence and intensity of GLUT1 expression [90]. The prognostic significance of GLUT1 expression was analyzed by immunohitochemistry in 67 patients with bone and soft-tissue sarcomas, and it was found that GLUT1 overexpression was significantly correlated with poor overall survival and with higher histological grade [91]. Therefore, GLUT1 overexpression could be used as a surrogate prognostic biomarker in patients with bone and soft-tissue sarcomas.

In endometrial cancer, the expression of GLUT1 and GLUT8 was analyzed in normal, atrophic, and malignant tissue. In normal endometrium and endometrial tumors, GLUT1 and GLUT8 were found to be expressed at distinct intracellular locations depending on the presence or absence of malignancy. In addition, GLUT1 upregulation was significantly associated with an increase of histological grade in endometrial tumors. Regarding to GLUT8, it was found an increase of expression in all endometrial tumor subtypes versus atrophic endometrium [92]. Therefore, since the expression of GLUT1 and GLUT8 increases during tumor progression and development of endometrial tumors, these GLUTs could be used as potential biomarkers for prognosis and clinical follow-up of endometrial cancer patients.

Finally, the expression of GLUT1, GLUT2, GLUT3, GLUT4, and GLUT10 was assayed by quantitative RT-PCR in 152 normal and pathological thyroid samples. Only GLUT1 showed a significant increase of expression in thyroid carcinoma versus normal tissue [93].

## 4. Glut Transporters and Anticancer Therapy

### 4.1. Pharmacological Modulation of Glucose Uptake by Current Anticancer Drugs

Nowadays, anticancer therapy is based on two main approaches. Firstly, the traditional approach is based on conventional chemotherapy addressed unspecifically against general cell processes, such as nucleotide biosynthesis. Secondly, novel approaches are based on the use of targeted therapy, which includes drugs designed to block specific components of signaling pathways deregulated in cancer. In this way, drugs as the multitargeted TKIs (tyrosine kinase inhibitors) sunitinib (Sutent, Pfizer) and sorafenib (Nexavar, Bayer), or temsirolimus (Torisel, Pfizer), an analog of rapamycin, inhibitor of the mTORC1 (mammalian target of rapamycin complex 1) formation, have contributed to substantial improvements in the treatment of patients affected of different tumors, such as for example RCC. However, probably due to the recent implantation of these targeted therapies, their effect on GLUT transporters remains, in many aspects, elusive. In a recent work [94], it was demonstrated that, in renal angiomyolipomas, which lack TSC1/TSC2 (tuberous sclerosis) complex and display a constitutive activation of mTORC1, the glucose uptake was surprisingly low. It is surprisingly because mTORC1 pathway was involved in the upregulation of glycolitic enzymes and GLUT transporters [95], as this upregulation is reached through the activation of HIF1A and VEGF signaling. The explanation for the low glucose uptake, under the absence of TSC1/2 complex and the mTORC1 constitutive activation, is that the trafficking to membrane of GLUT1, GLUT2, and GLUT4 proteins is impaired. This is important because the deregulation and activation of mTOR [mammalian target of rapamycin; official name: mechanistic target of rapamycin (serine/threonine kinase)] pathway may be behind of many tumor types. But, as it has been above described, tumors display an enhanced glucose uptake and GLUT transporter expression. Therefore, mTORC1 altered activity is insufficient to explain the increase of glucose uptake in tumors, and it is possible to hypothesize the existence of additional molecular events, beyond mTOR signaling, which contribute to enhance glucose consumption and metabolic hyperactivity during tumorigenesis. However, mTOR inhibitors (e.g., temsirolimus) are able to reduce glucose uptake in tumors [96] (e.g., kidney cancer), although this effect is probably related to the inhibition on tumor angiogenesis (inhibiting the mTOR-dependent HIF1/VEGF signaling) and glucose deprivation of tumor, rather than with a possible direct effect of mTOR pathway on GLUT expression and/or trafficking. In other recent study on an animal model, it was analyzed the utility of mTOR inhibitors (rapamycin) as therapeutic strategy to treat insulin-resistant states, including type 2 diabetes. This supposition is based on the fact that mTOR and its downstream S6K1 (ribosomal protein S6 kinase, 70 kDa, polypeptide 1) are able to downregulate IRS (insulin receptor substrate) proteins, with the consequent reduction of insulin-dependent signaling through PI3K/Akt (phosphoinositide 3-kinase/v-akt murine thymoma viral oncogene) pathway, GLUT4 translocation to plasma membrane, and glucose uptake. However, in this study, rapamycin appears to exacerbate diabetes, increasing the resistance to insulin, and reducing *β*-cell function in pancreas where it triggers apoptosis [97].

Regarding to TKIs (e.g., sunitinib), although still little is known about their effect on GLUT transporters in tumors, the antiangiogenic effect triggered by these drugs may be also involved in the reduction of glucose uptake, through the deprivation of tumor accessibility to glucose. However, it is feasible the existence of some direct effect of TKIs on GLUT expression and/or trafficking as there were detected side effects of these drugs, such as asthenia. In this way, some TKIs, as sunitinib, which is a multikinase inhibitor with a low specificity by its targets, may block multiple RTKs [such as INSR (insulin receptor) or IGFR1 (insulin-like growth factor 1 receptor)] or intermediate kinases (such as AMPK, PI3K, or Akt) in distinct signaling pathways, with direct action on glucose uptake or GLUT expression. Currently, there are several small molecules and antibodies under investigation designed against IGFR in different clinical trials. These new drugs probably may play some role on the expression and function of sugar transporters, since IGFR pathway is one of key pathways which controls sugar uptake in normal cells.

So far, selective GLUT inhibitors are not available in the clinical setting. Several selective GLUT inhibitors, such as fasentin or apigenin, have been tested *in vivo* and *in vitro*. These selective agents acts by blocking the glucose uptake from tumor cells and they have been shown to sensitize cells to death and avoid the normal activation of the PI3K/Akt/mTOR intracellular pathway [98, 99]. However, the bioavailability of these agents prevents the transition to clinical practice. 

### 4.2. Glucose Uptake Inhibition-Based Anticancer Therapies

As we have just reviewed, most tumors exhibit increased expression of sugar transporters as well as enhanced glycolysis. This phenomenon, which takes place even in aerobic conditions, that is, in presence of sufficient oxygen to carry out mitochondrial respiration, is known as Warburg effect [1], and is considered as a fundamental metabolic alteration during malignant transformation. There are several hypotheses to explain this phenomenon, such as mitochondrial malfunction, generation of a hypoxic tumor microenvironment, defects in oncogenic signaling, or metabolic abnormalities. In any case, the increased dependence of tumor cells on sugars and glycolytic pathway to generate ATP provides the biochemical basis to design drugs that preferentially kill cancer cells through the pharmacological inhibition of sugar transport and/or glycolysis.

The glycolytic inhibitors are particularly effective against tumors that display an increased glycolytic activity associated with mitochondrial defects or hypoxic conditions. These events are normally related to resistance and low response to conventional chemotherapy. Increased glycolysis is present in a wide spectrum of human tumors, and therefore the development of novel glycolytic inhibitors as anticancer agents would have broad therapeutic applications [100]. In this way, it has been recently presented a phase I trial to study the pharmacokinetic of 2-Deoxyglucose, a glycolytic inhibitor analog for glucose, to treat advanced solid tumors and hormone refractory prostate cancer [101]. This agent leads to sensitization of tumor cells to other pharmacological stimuli [102]. 

The inhibition of sugar transport may be reached through different approaches. One of these approaches is the use of antisense oligonucleotides against *GLUT* genes. This has been proved with GLUT5 which is expressed in breast cancer, but not in normal breast tissue [69, 70]. Two breast cancer cell lines, MCF-7, which is estrogen-receptor positive and mimics an early stage of breast cancer, and MDA-MB-231, which is estrogen-receptor negative and mimics a late stage in breast cancer progression, were exposed to a 15-nucleotide sequence around the start codon of *GLUT5*, used as an antisense oligonucleotide to specifically block GLUT5 expression. It was found that the oligonucleotide anti-*GLUT5* triggered antiproliferative effects on both breast cancer cell lines. This action, unlike current drugs used against breast cancer (e.g., tamoxifen), seemed to be specifically addressed on breast tumor cells (which express GLUT5) with independence of estrogen-receptor status [103]. An additional approach consists of the inhibition of cell sugar uptake. In this way, it has been demonstrated that D-allose, a rare sugar, is able to interfere with the D-glucose uptake and to induce apoptosis in head and neck tumor cells, inhibiting their growth [104].

## 5. Discussion

Sugars are the main substrate utilized by cells for energy production, so it is easily understandable their importance for life, both in normal cell physiology and in disease conditions. A key point to take into account in the study of sugar's metabolism is their introduction inside of cells. Sugars are moved through plasma membrane using carrier proteins, which are grouped in two main types: facilitative transporters, named GLUTs, and sodium-dependent transporters, named SGLTs. The fundamental difference between these two types of transporters is their dependence on energy usage to perform the transport: while the transport by GLUTs is performed through an energy-independent mechanism, the transport by SGLTs is energy-consuming. However, the basic structure of sugar transporters is similar. It is constituted by a number of transmembrane helix grouped to form a channel by which sugar crosses. Indeed, the similarity within each group goes beyond of structure, with important sequence identity between distinct members. The major difference between sugar transporters in each group is their main site of expression, since they show strong tissue specificity. For example, GLUT1 is often known as “erythroid” GLUT, as its expression is preponderant in erythrocytes. In fact, each GLUT carrier displays a number of particular features which are the suitable and essentials for energy requirements and proper function of the specific tissue where the GLUT is expressed. For example, GLUT4 is primarily expressed in insulin-sensitive tissues, where is able to translocate from an intracellular pool to plasma membrane [12–15]. Each tissue displays changes in energy requirements due to their particular physiology (e.g., skeletal muscle), so the glucose uptake must be adapted for these tissue specific requirements through internal or external stimuli directly related to tissue function, such as insulin or exercise. So, when a muscle is subjected to intense activity due to exercise, its glucose uptake must increase in order to assure the necessary energy supply and respond to the increase in glucose expenditure. This objective is achieved through a complex network of exercise-dependent signals which culminate with the mobilization of intracellular pool of GLUT4 to plasma membrane. Therefore, it is not surprising that alterations in the expression of GLUTs or their malfunction can trigger different diseases, such as diabetes. In tumors, alterations in GLUTs contribute to their maintenance and their virulence. These alterations in GLUTs lies in other background alterations which are behind different carcinogenetic events and affect simultaneously to multiple processes, as sugar uptake. For example, p53, a tumor suppressor protein with important functions in promoting apoptosis and cell cycle arrest when cell suffers any aggression or damage, is mutated or transcriptionally deregulated in many types of cancer. This is an event which is thought to drive many aspects of carcinogenesis. GLUT deregulation probably is among these aspects, as p53 is able to transcriptionally repress GLUT1 and GLUT4 gene expression [9]. Other signaling systems that directly affect to GLUT expression may be also altered in cancer, such as the PI3K/Akt/mTOR pathway which conveys the signal from insulin to cell. Moreover, carcinogenetic events which affect to EGFR may be behind the increased glucose uptake and survival of cancer cells through the stabilization of SGLT1 carrier, as it has been recently demonstrated [59]. Indeed, it was shown that *KRAS *activating mutations, occurring in different types of tumors, can be behind GLUT1 overexpression and also can be responsible for the increased glucose uptake in colorectal cancer cell lines [53]. All these alterations drive to an unfailing increase in sugar uptake which is one of hallmarks of cancer, such as it has been highlighted many years ago with the discovery of Warburg effect [1]. The increase in sugar uptake feeds to cancer cells in their expansive activity in which they burn huge energy amounts. This energy can be obtained through multiple ways, although the main system used by cancer cells is the glycolysis as these cancer cells normally display a number of physiological, metabolic, and genetic abnormalities that make impossible the mitochondrial oxidative phosphorylation. Glycolysis is an inefficient way to obtain energy which explains the vast necessity of sugar by cancer cells. In this way, there is some evidence showing the relationship between glucose transporter levels and prognosis in cancer. Therefore, it is easy to imagine distinct pharmacological approaches for the design of drugs to attack the feeding source of cancer cells. A first approach could be the blockade of signaling components altered in cancer that enhance the expression of sugar transporters, following with the current pharmacological design of multikinase inhibitors. Other strategies are the administration of glycolytic inhibitors, the direct blockade of sugar transporters, and the administration of nonmetabolizable sugar analogs. The use of glycolytic inhibitors has the disadvantage of their limited specificity, as they target any cell of organism. However, the use of nonmetabolizable sugar anologs is most plausible because cancer cells take up primarily glucose, whereas the remaining cells of the organism are able to efficiently metabolize a number of other sugars. Finally, the use of sugar carrier inhibitors is perhaps the most interesting option, as it is possible to design molecules that specifically inhibit each carrier protein. This could be used in the tumors where a sugar carrier is specifically expressed or where a particular sugar carrier shows an aberrant overexpression. The advantage of this approach is the relative overlapping of functions among sugar transporters (i.e., one carrier protein can transport distinct sugars with more or less affinity and, thus, to supply the lack of other), although their strong tissue-specific distribution is an issue to take into account. In any case, none of these pharmacological approaches are being currently explored. Current targeted therapies are not designed to decrease the sugar uptake by cancer cells, although this effect may be achieved as a secondary result. For example, sunitinib, a TKI used in the treatment of RCC and GIST (gastrointestinal stromal tumors), reached excellent results due to its antiangiogenic effect. The additional decrease in glucose uptake observed in cancer cells is probably due to the loss of tumor vasculature, with the consequent reduction in sugar supply. However, as asthenia is the most important side effect triggered by these TKIs, it is also possible some type of pharmacological modulation on the expression or function of sugar transporters. Undoubtedly, it is still necessary to carry out an important effort in understanding the role of sugar transporters and their deregulation in cancer, as well as to tackle the design of efficient drugs specifically targeted against the glucose uptake by cancer cells. In addition, prospectively designed trials measuring glucose transporter levels are needed to evaluate the potential role that these transporters may have in advanced cancer and their modulation by anticancer drugs. We think that GLUTs emerge as one of the key drivers on tumor cell growth and may represent a target for the development of new drugs.

## Figures and Tables

**Table 1:** Substrate specificity and tissue expression of sugar transporters.

Transporter	Substrates		Tissues									
	Glucose	Fructose	Intestine	Kidney	Blood	Liver	Brain	Pancreas	Testis	Muscle	Heart	Fat

SGLT1	**X**		**X**	**X**								

SGLT2	**X**			**X**								

GLUT1	**X**				**X**		**X**					

GLUT2	**X**		**X**	**X**		**X**		**X**				

GLUT3	**X**						**X**		**X**			

GLUT4	**X**									**X**	**X**	**X**

GLUT5		**X**	**X**						**X**	**X**		

GLUT6	**X**				**X**		**X**					

GLUT7	**X**	**X**	**X**						**X**			**X**

GLUT8	**X**						**X**		**X**			**X**

GLUT9		**X**		**X**		**X**						

GLUT10	**X**			**X**		**X**	**X**	**X**		**X**	**X**	

GLUT11	**X**	**X**		**X**						**X**	**X**	**X**

GLUT12	**X**		**X**							**X**		**X**

GLUT14	**X**								**X**			

**Table 2:** Sugar transporters and their expression in cancer.

Transporter	Tissues	Roles and properties
*SGLT transporters*		

SGLT1	Small intestine, kidney.	Intestinal absorption of glucose from meal. Renal reabsorption of glucose.
SGLT2	Kidney.	Renal absorption of glucose from glomerular filtrate.

*Class I GLUT transporters*		

GLUT1	Erythrocytes, brain (blood-brain barrier).	Basal glucose uptake.
GLUT2	Liver, pancreatic islet cells, small intestine, kidney.	Glucose sensing in pancreatic *β*-cells. Trans-epithelial glucose and fructose transport. High-capacity, low-affinity glucose transporter.
GLUT3	Brain (neuronal), testis.	Glucose neural transporter.
GLUT4	Muscle, heart, adipose tissue.	Expressed in tissues with insulin-stimulated acute glucose transport. In response to insulin, it is translocated to plasma membrane.
GLUT14	Testis.	

*Class II GLUT transporters*		

GLUT5	Small intestine, testis, muscle.	Only fructose transporter.
GLUT7	Intestine, testis, prostate.	
GLUT9	Liver, kidney.	
GLUT11	Heart, adipose tissue, kidney, placenta, muscle.	GLUT11 has three isoforms: GLUT11a, GLUT11b, and GLUT11c, with distinct tissue distribution.

*Class III GLUT transporters*		

GLUT6	Brain, spleen, leukocytes.	
GLUT8	Brain, testis, adipocytes.	
GLUT10	Heart, lung, brain, liver, skeletal muscle, pancreas, placenta, and kidney.	Mutations in GLUT10 were associated with arterial tortuosity syndrome. GLUT10 deficiency is associated with the upregulation of TGFB pathway in Loeys-Dietz syndrome.
GLUT12	Placenta, adipose tissue, small intestine and skeletal muscle.	In skeletal muscle, it is translocated to plasma membrane in response to insulin, like GLUT4.
HMIT	Brain.	Myoinositol transporter.
